# Evaluation of Substance Use Disorder Readmission and Length of Hospital Stay in a Major Rehabilitation Center in the Gulf States: a Retrospective Cohort Study

**DOI:** 10.1007/s11469-022-00920-z

**Published:** 2022-09-29

**Authors:** Majed Ramadan, Aysha Alharbi, Rami Ghazi Ahmad, Ahmed Alkhalaf, Noara Alhusseini, Alanood S. Algarni, Izzeldin Siddig Mohamed

**Affiliations:** 1 Population Health Research Section, King Abdullah International Medical Research Center (KAIMRC), King Saud Bin Abdulaziz University for Health Sciences, Ministry of National Guard - Health Affairs, P.O.BOX 9515, Jeddah, 21423 Kingdom of Saudi Arabia; 2 Ministry of Health, Prince Abdulrahman Bin Abdulaziz St., Riyadh, Kingdom of Saudi Arabia; 3 Psychiatry Section, Medicine Department, Ministry of National Guard - Health Affairs, Abdullah International Medical Research Center, Jeddah, 21423 Saudi Arabia; 4 Psychiatry & Mental Health Services, Johns Hopkins Aramco Healthcare, Dhahran, Kingdom of Saudi Arabia; 5 Department of Biostatistics and Epidemiology, College of Medicine, Alfaisal University, Riyadh, Saudi Arabia; 6 Pharmacology and Toxicology Department, College of Pharmacy, Umm Al-Qura University, Mecca, Saudi Arabia; 7 Quality Department, Al-Amal Hospital, Jeddah, 21472 Saudi Arabia

**Keywords:** Substance use disorder, Readmission, Amphetamine, Hospitalization, Stimulants

## Abstract

Substance use disorders (SUDs) patients have longer lengths of hospital stay, and more unplanned readmissions than other hospitalized patients. We aim to evaluate SUD-related rehospitalization and length of hospital stay in a major rehabilitation center that serves countries of the Gulf States. In a retrospective cohort study for 16-year data set in Al-Amal Hospital Electronic Health Record in the city of Dammam, Eastern region of Saudi Arabia, patients received services from the SUD treatment programs in the period of January 1, 2005, to December 31, 2021. We used cause-specific Cox proportional hazards regression model to estimate risk of readmission, and general linear model to examine the association between substance use disorders and length of hospital stay. Of the total cohort, 4398 (30.17%) were readmitted within 1 year of discharge date. More than half of the cohort were unemployed patients (52.93%). Patients diagnosed with amphetamine use disorder were 1.36 higher risk of readmission compared to no amphetamine disorder (HR = 1.36; CI (1.04, 1.78) *P*.02). Patients diagnosed with mental disorder had 7.25 times higher risk of longer hospital stay compared to no mental health disorder (coefficient = 7.25; *P* < .0001). Amphetamine use disorder increased the risk of readmission. A secondary diagnosis of mental disorders among SUD patients increased length of hospital stay. As a targeted region of amphetamine smuggling in the world, policy and clinical decision-makers in Saudi Arabia and the Gulf States should consider taking proactive steps to minimize the future anticipated high demand for addiction treatment in the region.

Substance use disorders (SUDs) remain the leading cause of disability and premature mortality, impacting health, social care, welfare, and criminal justice systems worldwide (Blackwood et al., 2021; Whiteford et al., 2016). According to a recent report by the World Health Organization, excessive alcohol consumption results in approximately three million deaths annually worldwide (World Health Organization, 2020). From an epidemiological perspective, stimulants are the second most commonly used drugs worldwide, with an estimated 68 million past-year users (World Health Organization, 2020). However, the types of stimulants employed vary markedly across countries and regions worldwide (Hurst, 2019). In the Middle East, amphetamine is the most widely used illicit drug (Hurst, 2019). Between 2013 and 2017, the main destination market for amphetamine smuggled to the Middle East was Saudi Arabia, followed by Gulf countries (the United Arab Emirates, Qatar, Kuwait, and Bahrain) (Hurst, 2019). According to a World Drug Report, Saudi Arabia reportedly seized the largest quantities of amphetamine globally, accounting for a quarter of the total quantity seized worldwide during 2013–2017 (Hurst, 2019), potentially increasing the number of users and SUD individuals seeking treatment. Accumulating evidence has demonstrated that the striking increase in the prevalence rates of substance use or psychotic experience is a key player in the development of certain types of SUDs (Degenhardt et al., 2018; Kuepper et al., 2011).

Studies on epidemiology, treatment, and rehabilitation of SUD are scarce in the Middle East (Saquib et al., 2020). The absence of national surveillance systems that can track patterns and changes in nationwide substance use and related hospitalizations hinders understanding the needs of this vulnerable population. However, in Saudi Arabia and the Gulf States, the burden of SUDs is anticipated to be substantial (AbuMadini et al., 2008; Saquib et al., 2020). More individuals with drug-related SUDs have been seeking treatment in the last decade (Ibrahim et al., 2018). Nevertheless, patients with SUD tend to overutilize hospital and emergency department services (Nordeck et al., 2018). Indeed, individuals with SUDs have a longer length of hospital stay and more unplanned readmissions than the general population of hospitalized patients (Liebschutz et al., 2014; Nordeck et al., 2018). Furthermore, the cost of unplanned readmissions is higher than that of planned admissions (Sandhu, 2011). Nearly 3.3 million (58.2%) readmissions were reported within 30 days of the original discharge in the USA, accounting for more than half of the total annual cost of $41.3 billion (Englander et al., 2017). Hence, it is imperative to assess the probability of readmission for specific groups of patients and diseases. Conversely, the length of hospitalization is one of the highest among patients with SUDs (Hines et al., 2014). In the USA, SUD length of stay accounted for approximately 7% of all stays (Heslin et al., 2015), with an average of 6-day stay in non-psychiatric hospitals and 36 days in psychiatric hospitals (Crossley et al., 2020). Moreover, patients with a prolonged length of stay are at an increased risk of acquiring hospital-related infections (Lyketsos et al., 2002). Therefore, evaluating factors contributing to readmissions and length of hospital stay would better address the specific needs of the population at risk and prevent costly inpatient treatment (Ahmedani et al., 2015; Lyketsos et al., 2002).

Given these findings, the strain on the healthcare system and cost of care are inevitable. In contrast, SUDs and mental health admissions present the highest percentage of potentially preventable readmissions (Ahmedani et al., 2015). Several hospitals have formed specialized mental health and SUD consultation-liaison services to potentially reduce the escalating readmission rates (Gillies et al., 2015). However, effective consultation services or other prevention strategies warrant in-depth evaluation of institutions that provide the required services. Thus, given the importance of improving SUD-related services and avoiding the burden that can result from readmissions and hospital length of stay, we aimed to evaluate substance use disorders related to rehospitalization and length of hospital stay in a major rehabilitation center that serves six countries in the Gulf States.

## Methods

### Study Design, Setting, and Participants

**Fig. 1:**
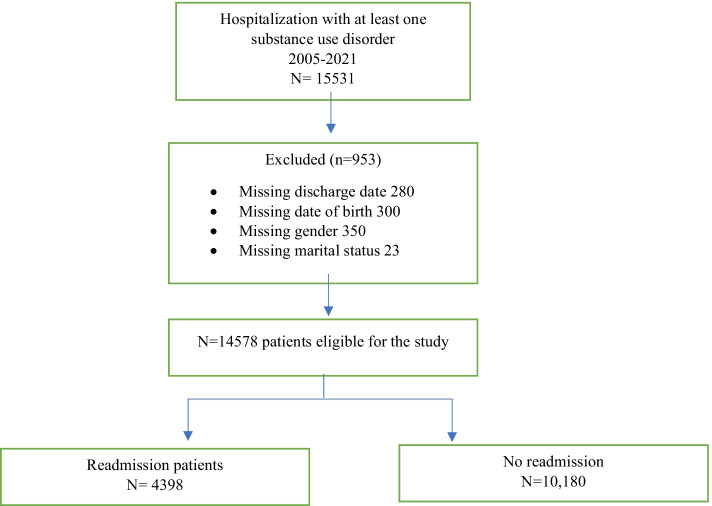
Exclusion criteria diagram

This retrospective cohort study was performed using the Electronic Health Record System of Al-Amal Hospital, Dammam, Saudi Arabia, including all patients who received services from the SUD treatment programs from January 1, 2005, to December 31, 2021. Al-Amal Hospital is a major addiction and rehabilitation center in the region that operates under the Ministry of Health and provides free treatment and rehabilitation services. The hospital adapted the 12 Steps Program to assist their patients in addiction recovery (Donovan et al., 2013). The 12 Steps Program was started after the establishment of the center. The population of Al-Amal Hospital includes individuals residing in the Saudi Arabian region and Gulf States (United Arab Emirates, Qatar, Kuwait, Oman, and Bahrain). Individuals aged ≥ 12 years, who were diagnosed with an SUD, substance dependence, or substance abuse and hospitalized between 2005 and 2021, were included in the present study. After applying the exclusion criteria, 14,505 hospitalized patients were eligible for study inclusion. In total, 953 individuals were excluded from the study owing to missing discharge dates, dates of birth, sex, and marital status (Fig. 1). This cohort study was approved by the Ministry of Health Institution Review Board, and the requirement for informed consent was waived because the data were anonymized.

### Study Variables and Data Source

#### Demographic Variables

Patient demographics included age, sex, marital status, nationality, employment status, discharge status, year of admission, and country of residence. The authors included the following comorbidities as confounding factors: human immunodeficiency virus, hepatitis B virus, hepatitis C virus, hypertension, diabetes, and mental health disorders (including depression, anxiety, personality disorder, schizophrenia, antisocial, and substance-induced psychosis). Diagnoses were extracted from the health record system using the International Classification of Diseases (ICD-9-CM and ICD-10-CM codes) for each admission and then confirmed with written clinical primary and secondary diagnoses.

#### Outcome Variables

Herein, the main outcome variable was hospital readmission within 1 year of discharge date (dichotomized: hospital readmission within the study period *vs*. no hospital readmission within the study period), given that the implemented treatment, the 12 Steps Program, at the hospital requires an average of 30 days of hospital stay. Patients with a history of at least one admission within the study period from 2005 to 2021 were categorized as readmitted, and those with no previous admission history within the study period from 2005 to 2021 were categorized as having no hospital readmission. The follow-up time started on the date of admission and ended on the last discharge date. The application of this follow-up plan allowed us to account for the longest potential follow-up duration covering the entire study period. Patients with missing discharge data were excluded from the study (Fig. 1). Completeness of follow-up was computed at each time interval using Clark’s completeness index (CCI) and a simplified person-time (SPT) method (Bursac et al., 2008; Clark et al., 2002; von Allmen et al., 2015; Xue et al., 2017). The resulting CCI and SPT values were 80% and 81%, respectively. Patients diagnosed with one of the following SUD (DSM-IV abuse or dependence) served as independent variables: hashish/marijuana/cannabis dependence, alcohol dependence, opioids (tramadol, heroin, codeine, hydrocodeine, hydromorphine, opium, pethidine, and morphine), amphetamine/m*ethamphetamine* dependence, polysubstance abuse, benzodiazepines (Xanax), *pregabalin* (Lyrica), and volatile inhalants. Specific ICD9 and 10 codes are assigned for polysubstance abuse disorders. Individuals diagnosed with polysubstance abuse disorders will not be diagnosed with other SUDs. Independent variables were dichotomized (yes *vs*. no). Each SUD was examined separately as an independent, mutually exclusive event.

### Statistical Analysis

For data analyses, all patients with SUDs were classified into two groups: those with and without readmission within 1 year (365 days) after discharge. The demographic and hospital-related characteristics and SUD diagnoses were assessed across frequent SUD readmissions and non-readmission patients, using unadjusted chi-square, Fisher exact, and independent *t*-tests, where appropriate. We used purposeful variable selection (Clark et al., 2002) for model building in multivariate analyses. Backward elimination was used to retain all variables with *P* < 0.3. The time to last readmission rate was estimated using the Kaplan–Meier product-limit method, and differences between curves were assessed using the log-rank test for specific-cause SUD readmission. We used a cause-specific Cox proportional hazards regression model to estimate the risk of readmission in patients diagnosed with SUDs. Furthermore, we assessed the proportional hazard assumption by using Schoenfeld residuals. The Cox proportional hazards regression analysis included only significant predictors from the variable selection. We used a general linear model (GLM) to examine the association between SUDs and length of hospital stay in days adjusted for covariates. The assumptions of normality and homogeneity of variance were also assessed. All statistical tests were 2-sided, and the findings were considered statistically significant at *P* < 0.05. All analyses were conducted using the SAS statistical software (version 9.4; SAS Institute Inc. Cary, NC).

## Results

### Demographic, Social, and Economic Factors

After applying exclusion criteria, between January 2005 to December 2021, 14,578 patients were admitted to Al-Amal Hospital in Dammam. Of these, 4398 (30.17%) were readmitted to the hospital within 1 year of discharge date. The cohort predominantly included males 14,535 (99.7%) with mean age of 39 years old, single 8961 (61.47%), and unemployed 7716 (52.93%) (Table 1). Average length of stay was slightly higher among patients with readmission status (25.6, 25.5 days, respectively) (*p* 0.3) with mean of 3 readmission times during the study period. The cohort was predominantly Saudis 13,789 (94.59%) followed by Kuwait’s (1.78%), and Omani’s nationalities (1.56%) (Table 1). There was a statistically significant difference in discharge status and readmission to the hospital (*p* 0.03). About 1 out of 4 patients discharged with family request was readmitted to the hospital (Table 1). Year of admission (2011 to 2016) (38.36%) tends to have the highest readmission rate compared to 2005–2010 and 2017–2021 (34.95%, 26.69%, respectively).

**Table 1:** Demographic characteristics of patients admitted to Al-Amal Hospital (2005–2021)

	Total *N* (%)	Readmission *n* (%)	No readmission *n* (%)	*P*^1^
Total	14,578 (100)	4398 (30.17)	10,180 (69.83)	
Age				< .0001 ^2^
Mean (SD)	39 (10.47)	40 (10.35)	38 (10.49)	
Gender				.74
Male	14,535 (99.7)	4386 (99.73)	10,149 (99.7)	
Female	43 (0.3)	12 (0.27)	31 (0.3)	
Marital status				.43
Married	4436 (30.43)	1380 (31.38)	3056 (30.02)	
Single	8961 (61.47)	2668 (60.66)	6293 (61.82)	
Divorced	1156 (7.93)	343 (7.8)	813 (7.99)	
Widow	25 (0.17)	7 (0.16)	18 (0.18)	
Employment status				.77
Employed	504 (3.46)	153 (3.48)	351 (3.45)	
Employment (government)	3564 (24.45)	1040 (23.65)	2524 (24.79)	
Employment (private sector)	1296 (8.89)	392 (8.91)	904 (8.88)	
Student	599 (4.11)	180 (4.09)	419 (5.29)	
Retired	763 (5.23)	224 (5.09)	539 (5.29)	
Unemployment	7716 (52.93)	2363 (53.73)	5353 (52.58)	
Unknown	136 (0.93)	46 (1.05)	90 (0.88)	
Length of stay per day				.3
Mean (median)	27.37 (26)	25.6 (25)	25.5 (26)	
Number of readmissions				
Mean (SD)	3 (3.08)	3(3.08)	0(0)	
Country of resident				.06
Saudi Arabia	13,789 (94.59)	4136 (94.04)	9653 (94.82)	
United Arab Emirates	6 (0.04)	1 (0.02)	5 (0.05)	
Bahrain	152 (1.04)	62 (1.41)	90 (0.88)	
Kuwait	260 (1.78)	73 (1.66)	187 (1.84)	
Oman	228 (1.56)	82 (1.86)	146 (1.43)	
Qatar	29 (0.2)	9 (0.2)	20 (0.20)	
Other	114 (0.77)	35 (0.77)	79 (0.78)	
Discharge status				.003
Recovered	11126 (76.32)	3328 (75.67)	7798 (76.6)	
Against medical advice	2414 (16.56)	710 (16.14)	1704 (16.74)	
Family request	379 (2.6)	149 (3.39)	230 (2.26)	
No show up after break	135 (0.93)	45 (1.02)	90 (0.88)	
Not fit for the program	60 (0.41)	23 (0.52)	37 (0.36)	
Police request	404 (2.77)	128 (2.91)	276 (2.71)	
Transfer to another hospital	60 (0.41)	15 (0.34)	45 (0.44)	
Year of admission				< .0001
2005–2010	3744 (25.68)	1537 (34.95)	2207 (21.68)	
2011–2016	5537 (37.98)	1687 (38.36)	3850 (37.82)	
2017–2021	5297 (36.34)	1174 (26.69)	4123 (40.50)	

^1^Chi-square and fisher exact test when appropriate

^2^ T-test *p*-value

### Characteristics of SUD Readmission

In the present cohort, 6 of 10 admitted patients had at least one cannabis use disorder diagnosis 8785 (60.26%), followed by alcohol (31.29%) and opioid (15.2%) use disorders. However, we detected no significant difference in the readmission rate among patients diagnosed with cannabis use disorder (*P* = 0.42) (Table 2). There was a statistically significant difference in readmission rates among alcohol use disorder (*P* < 0.0001), opioid disorder (*P* < 0.001), amphetamine/m*ethamphetamine* dependence (*P* < 0.001), benzodiazepines (*P* < 0.04), and *pregabalin use* (*P* < 0.05). In addition, the admitted patient cohort presented with mental health disorders (12.5%) and comorbidities (0.8%) (Table 2).

**Table 2:** Substance use disorder for patients admitted to Al-Amal Hospital (2005–2021)

	Total	Readmission	No readmission	*P*^1^
	*N* (%)	*n* (%)	*n* (%)	
	14,578 (100)	4398 (30.17)	10,180 (69.83)	
Hashish/marijuana/cannabis dependence				.42
Yes	8785 (60.26)	2672 (60.75)	6113 (60.05)	
No	5793 (39.74)	1726 (39.25)	4067 (39.95)	
Alcohol dependence				< .0001
Yes	4562 (31.29)	1482 (33.7)	3080 (30.26)	
No	10,016 (68.71)	2916 (66.3)	7100 (69.74)	
Opioid (tramadol, heroin, codeine, hydrocodeine, hydromorphine, opium, pethidine, morphine) dependence				.001
Yes	2216 (15.2)	731 (16.62)	1485 (14.59)	
No	12,362 (84.8)	3667 (83.38)	8695 (85.41)	
Amphetamine/m*ethamphetamine* dependence				.001
Yes	1411 (9.68)	372 (8.46)	1039 (10.21)	
No	13,167 (90.32)	4026 (91.54)	9141 (89.79)	
Poly substance abuse/dependence				.008
Yes	1287 (8.83)	430 (9.78)	857 (8.42)	
No	13,291 (91.17)	3968 (90.22)	9323 (91.58)	
Benzodiazepines (Xanax) dependence				.04
Yes	1390 (9.53)	452 (10.28)	938 (9.21)	
No	13,188 (90.47)	3946 (89.72)	9242 (90.79)	
*Pregabalin* (Lyrica) dependence				.005
Yes	364 (2.5)	86 (1.96)	278 (2.73)	
No	14,214 (97.5)	4312 (98.04)	9902 (97.27)	
Volatile inhalant dependence				.08
Yes	188 (1.29)	46 (1.05)	142 (1.39)	
No	14,390 (98.71)	4352 (98.95)	10,038 (98.61)	
Mental health disorder (depression, anxiety, personality disorder, schizophrenia, antisocial, substance-induced psychosis)				< .0001
Yes	1822 (12.5)	460 (10.46)	1362 (13.38)	
No	12,756 (87.5)	3938 (89.54)	8818 (86.62)	
Comorbidity (HIV, HBV, HCV, hypertension, diabetes)				.41
Yes	116 (0.8)	39 (0.89)	77 (0.76)	
No	14,462 (99.2)	4359 (99.11)	10,103 (99.24)	

^1^Chi-square test

#### Risk of Readmission

**Fig. 2:**
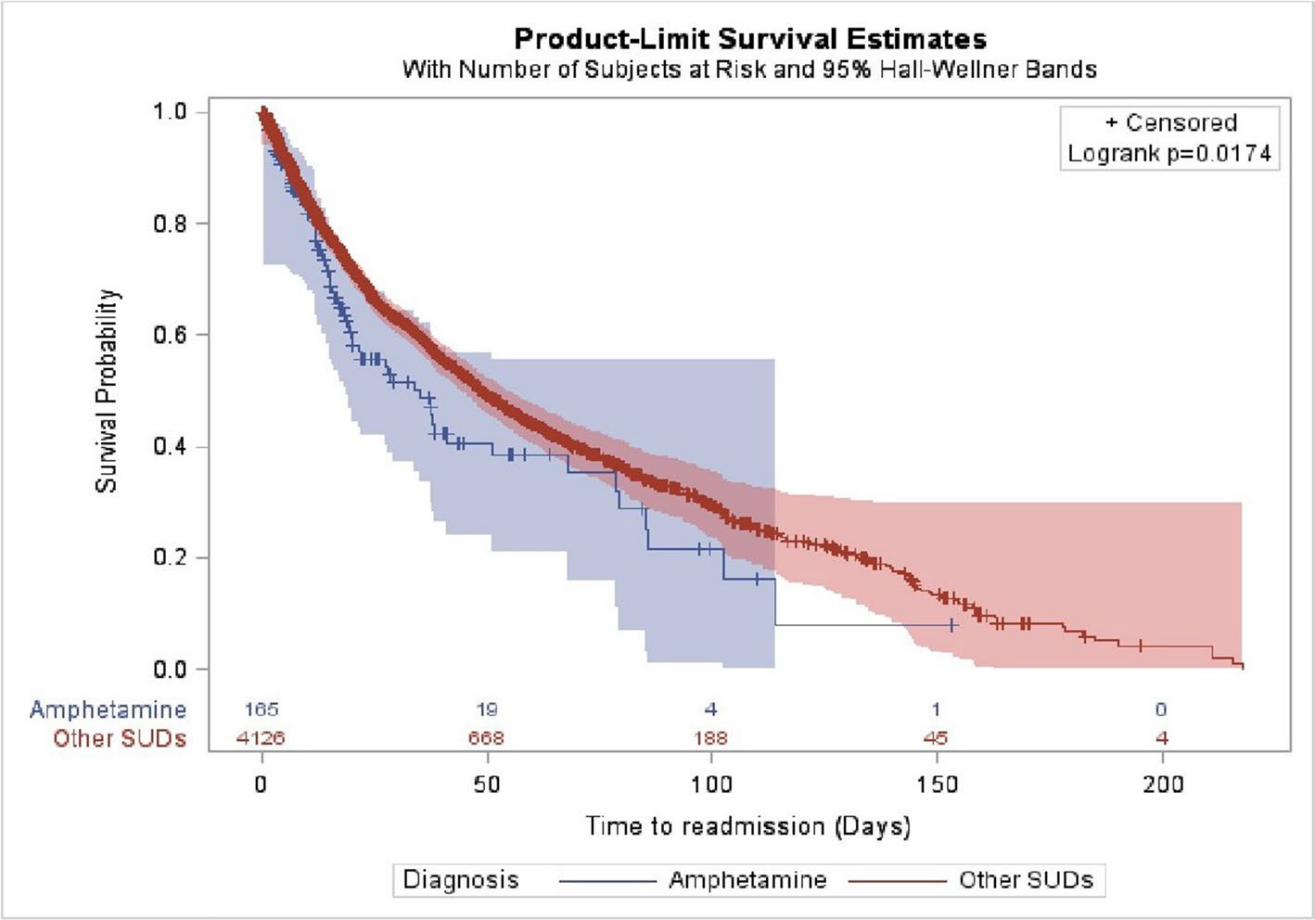
Time to first readmission curve

In the Cox regression analyses, there were no demographic factors associated with risk or readmission except for the year of admission. Those admitted between 2011 and 2016 years were at a higher risk of readmission compared to 2005–2010 years of admission (HR = 1.54, CI (1.31, 1.79), *P* < 0.0001) (Table 3). Amphetamine/methamphetamine use disorder was the only type of substance use disorder statistically significant with risk of readmission (HR = 1.36; CI (1.04, 1.78), *P*.02). Patients diagnosed with amphetamine/methamphetamine use disorder had a 1.36 higher risk of readmission than those with no amphetamine/methamphetamine use disorder (Table 4, Fig. 2). In Kaplan–Meier estimator graph, patients diagnosed with amphetamine/methamphetamine use disorder have a shorter time to readmission than other substance use disorders (Log-Rank *p* = 0.017) (Fig. 2).

**Table 3:** Risk of readmission and length of hospital stay demographic predictors among patients diagnosed with substance use disorder

	Risk of readmission			Length of hospital stay predictors		
	Hazard ratio (HR)	95% CI^1^	*P* value	Regression coefficient (β)	Standard error	*P*
Age						
12–25	0.82	(0.26, 2.58)	.74	-0.46	0.86	.59
26–40	Reference	Reference	Reference	1.22	0.71	.08
41–60	1.08	**(**0.97, 1.21)	.13	1.39	0.66	.03
61–112	0.88	**(**0.69, 1.12)	.31	Reference	Reference	Reference
Gender						
Male	**–––**^3^	**–––**	**–––**	Reference	Reference	Reference
Female	**–––**	**–––**	**–––**	0.61	2.14	.77
Marital status						
Married	1.44	**(**0.2 10.37)	.71	0.85	2.79	.31
Single	1.44	**(**0.2, 10.35)	.71	2.9	2.79	.29
Divorced	1.47	**(**0.2, 10.63)	.69	2.14	2.81	.76
Widow	Reference	Reference	Reference	Reference	Reference	Reference
Employment status						
Employed				1.07	0.68	.11
Employment (government)	**–––**	**–––**	**–––**	Reference	Reference	Reference
Employment (private sector)	**–––**	**–––**	**–––**	1.98	0.48	< .0001
Student	**–––**	**–––**	**–––**	2.16	0.65	.001
Retired	**–––**	**–––**	**–––**	4.19	0.61	< .0001
Unemployment	**–––**	**–––**	**–––**	2.16	0.33	< .0001
Unknown	**–––**	**–––**	**–––**	1.27	1.23	.29
Discharge status						
Recovered	Reference	Reference	Reference	8.25	0.71	< .0001
Against medical advice	0.98	**(**0.83, 1.15)	.83	− 8.0	0.75	< .0001
Family request	0.97	**(**0.8, 1.77)	.76	− 2.16	1.01	.03
No show up after break	1.11	(0.75, 1.62)	.59	− 0.71	1.38	.61
Not fit for the program	1.32	**(**0.72, 2.4)	.36	− 8.33	1.92	< .0001
Police request	0.85	(0.53, 1.39)	.53	Reference	Reference	Reference
Transfer to another hospital	0.76	**(**0.28, 2.05)	.60	− 9.98	1.92	< .0001
Year of admission						
2005–2010	Reference	Reference		Reference	Reference	
2011–2016	1.54	**(**1.31, 1.79)	< .0001	2.57	0.31	< .0001
2017–2021	**–––**^**2**^	**–––**	–––	1.87	0.34	< .0001

^1^Confidence interval (CI)

^2^No discharge date beyond 2021

^3^Removed by selection forward (not significant predictor)

**Table 4:** Risk of readmission and length of hospital stay predictors among patients diagnosed with substance use disorder

	Risk of readmission			Length of hospital stay predictors		
	Hazard ratio (HR)	95% CI^1^	*P* value	Regression coefficient (β)	Standard error	*P*
Hashish/marijuana/cannabis dependence	0.97	**(**0.84, 1.12)	.72	− 0.41	0.33	.22
Alcohol dependence	1.15	**(**0.94, 1.41)	.14	− 1.78	0.52	.0006
Opioid (tramadol, heroin, codeine, hydrocodeine, hydromorphine, opium, pethidine, morphine)	0.89	**(**0.69, 1.15)	.39	− 4.53	0.59	< .0001
Amphetamine/m*ethamphetamine* dependence	1.36	**(**1.04, 1.78)	.02	− 0.54	0.57	.33
Poly substance abuse/dependence	1.29	**(**0.79, 2.09)	.3	− 3.71	1.66	.02
Benzodiazepines (Xanax) dependence	0.87	**(**0.72, 1.05)	.15	− 1.12	0.49	.02
*Pregabalin* (Lyrica) dependence	0.91	(0.63, 1.91)	.65	− 1.14	0.84	.17
Volatile inhalant dependence	0.59	**(**0.27. 1.25)	.17	0.05	1.81	.97
No specified substance use disorder	Reference	Reference	Reference	Reference	Reference	Reference
Mental health disorder (depression, anxiety, personality disorder, schizophrenia, antisocial, substance-induced psychosis)						
Yes	0.75	**(**0.51, 1.11)	.15	7.25	0.67	< .0001
No	Reference	Reference	Reference	Reference	Reference	
Comorbidity (HIV, HBV, HCV, hypertension, diabetes)						
Yes	1.13	**(**0.72, 1.75)	.58	− 3.19	1.31	.01
No	Reference	Reference	Reference	Reference	Reference	

^1^Confidence interval (CI)

### Length of Hospital Stay Predictors

In the GLM analysis, several demographic factors were associated with the length of hospital stay. Compared with the 61–112 age group, the 41–60 age group was significantly associated with the length of hospital stay (*P* = 0.03). The 41–60 age group had a 1.39-fold greater mean length of hospital stay than the 61–112 age group (Table 3). The same pattern was observed in terms of employment status; retired patients tended to exhibit a 4.19-fold higher risk of longer hospital stay than employed patients (regression coefficient = 4.19; *P* < 0.0001) (Table 3). Patients with recovered discharge status presented an 8.25-fold higher risk of prolonged hospital stay than those with police request discharge status (regression coefficient = 8.25; *P* < 0.0001). Patients diagnosed with mental health disorders had a 7.25-fold higher risk of longer hospital stay than those not diagnosed with mental health disorders (regression coefficient = 7.25; *P* < 0.0001) (Table 4).

## Discussion

In the present study, we aimed to examine potential factors associated with readmission and length of hospital stay among patients with SUDs admitted to the Al-Amal Hospital. Our study recorded that 3 of 10 patients with SUDs were readmitted to the hospital, consistent with previous studies conducted in Europe (Böckmann et al., 2019) and the USA (Rowell-Cunsolo et al., 2020), where the average readmission rates ranged between 25 and 42%. Similarly, in our study, the average age of readmitted patients was 40 years, in line with the findings of a Swiss study (Böckmann et al., 2019). Consistent with previous studies, our study found that more than half of the readmitted patients were unemployed or retired, whereas the average number of readmitted unemployed patients ranged between 41 and 55% in previous studies (Böckmann et al., 2019; Laudet, 2012). However, this finding should be interpreted with caution, as whether the admitted patients lost their job before or after developing SUDs could not be determined using the current retrospective data. Interestingly, no other demographic factors were associated with the risk of readmission or length of hospital stay, except for employment status.

In the current study, unemployed and retired patients had a higher mean readmission rate than employed patients. Unemployment is a constant challenge among patients with SUD and has been long documented in the available literature (Bray et al., 2000; Laudet, 2012). Most previous studies have reported that nearly half of the SUD population was unemployed (Laudet, 2012; Bray et al., 2000; Hogue et al., 2010). Including nationally representative data, the Drug and Alcohol Services Information System has documented low employment rates among adult individuals undergoing SUD treatment: less than one-third of the sample (31%) were employed (Substance Abuse and Mental Health Services Administration Office of Applied Studies, 2008). A recent experimental study on the economic condition and admission among patients with SUD has concluded that economic hardship may increase the number of SUD admissions (Azagba et al., 2021). The coronavirus disease (COVID-19) pandemic resulted in economic downturns worldwide, including in Saudi Arabia. The economic burden prevents job seekers, especially vulnerable populations such as those with SUDs, from obtaining a job and maintaining a stable economic status.

In the present study, the 41–60 age group was significantly associated with a longer hospital stay than the older group (61–112). As suggested in previous studies, patients with SUD tend to be younger (46 years old) than those admitted for conditions other than SUDs (60 to 80 years old) (Bursac et al., 2008). Our study indicates that patients with SUDs with recovered discharge status exhibited a higher mean length of hospital stay, potentially indicating that completing the treatment program requires a longer hospital stay. In the present study, the average length of stay for all patients with SUD was 27 days, which is relatively less than that reported in a prior study conducted in acute psychiatric wards in the West, with an average hospital stay of 36 days (Crossley et al., 2020). The long hospital stay can be attributed to the 12-step treatment program established et al.-Amal Hospital, which requires the patient to be admitted for 30 days. This highlights the need to reevaluate the current treatment program and its efficacy on patient outcomes as factors associated with how SUD services impact the length of stay (Crossley et al., 2020). Furthermore, mental health disorders were another predictor of the length of hospital stay. It is well-documented that long-term and heavy users of SUDs might develop psychiatric disorders at some point in their lives (Winkelman et al., 2018). Factors that increase the severity of illness as a psychiatric disorder have been shown to increase the length of stay (Crossley et al., 2020). Thus, in the present study, SUD patients with mental health disorders as a second diagnosis appeared to spend more hospital days than those without mental health disorders.

Our study has several limitations that need to be addressed. First, the retrospective nature of the study included an uncontrolled methodology with potential selection bias and a limited number of patients from a single cohort. Thus, our findings should be validated in a larger prospective study to better represent the Kingdom of Saudi Arabia and the Gulf States and minimize potential selection bias. Second, factors associated with readmission and length of hospital stay, such as a history of mental illness, were unavailable in the hospital data for the study period. These data would have allowed an additional evaluation of readmission and length of hospital stay. Third, the data included an extremely small sample of female participants, which might have induced a gender bias. However, it is important to note that the hospital was designed for male services only until 2012, following which a new female section was established.

## Conclusion

In the current retrospective cohort study, our results reinforce findings from previous studies in the West, considering factors associated with readmission and length of hospital stay in patients with SUD. We demonstrated that employment status is a significant predictor of SUD-related readmission. As reported previously, we found that patients with amphetamine use disorder are at a higher risk of readmission than patients with other SUD. A secondary diagnosis of mental health disorders among patients with SUD could increase the length of hospital stay. Identifying populations at risk of high healthcare utilization remains crucial. This can help inform discharge planning and develop programs and interventions designed to improve health outcomes among this population. As a targeted region for global amphetamine smuggling, policy and clinical decision-makers in Saudi Arabia and the Gulf States should consider taking proactive steps to develop programs that evaluate the current treatment programs and minimize the anticipated high demand of addiction treatment in the region.

## Data Availability

The data that support the findings of this study are available from [Al-Amal Hospital, and Ministry of Health]. Restrictions apply to the availability of these data, which were used under license for this study. Data are available [at https://www.moh.gov.sa/en/Ministry/Forms/Studies-and-Researches/Pages/default.aspx] with the permission of [Ministry of Health].
